# The diagnostic dilemma of a gallbladder volvulus: An unusual case report and review of the literature

**DOI:** 10.1016/j.ijscr.2021.01.108

**Published:** 2021-02-03

**Authors:** Zarrukh Baig, Vladimir Ljubojevic, Francis Christian

**Affiliations:** a Department of Surgery, University of Saskatchewan, Saskatoon, Canada; b Department of Radiology, University of Saskatchewan, Saskatoon, Canada; c Professor of Surgery, University of Saskatchewan, Saskatoon, Canada

**Keywords:** Gallbladder volvulus, Torsion, Cholecystectomy, Hepatobiliary iminodiacetic acid (HIDA) scan

## Abstract

**Introduction and importance:**

A gallbladder volvulus is a rare medical condition requiring emergency surgery. There are 500 cases reported in the literature, and only 10 % have ever been diagnosed preoperatively. Gallbladder volvulus occurs when the gallbladder torts around the cystic duct and cystic artery resulting in occlusion of both structures and consequently, ischemia of the gallbladder. The diagnosis is challenging because the symptoms mimic cholecystitis without distinct radiological features specific for a volvulus.

**Case presentation:**

In this article, we report the case of a 77-year-old female who underwent ultrasonography (US), computed tomography (CT), magnetic resonance cholangiopancreatography (MRCP), and cholescintigraphy, which all reported features of cholecystitis. She underwent a cholecystectomy on admission day 5 because of refractory pain despite treatment with intravenous antibiotics. Intraoperatively, she was discovered to have complete gallbladder torsion with gangrene. Post-operatively, she had immediate and complete resolution of pain, and made a rapid recovery.

**Clinical discussion:**

We review the available literature to determine radiological characteristics specific to a gallbladder volvulus. Patients without cholelithiasis and incomplete filling of the gallbladder in a nuclear medicine scan should be evaluated for gallbladder volvulus.

**Conclusion:**

Through this report, we suggest a high index of suspicion for gallbladder volvulus in elderly female patients with signs and symptoms of acalculous cholecystitis that have no resolution in symptoms with conservative management.

## Highlights

• Gallbladder volvulus is a challenging radiological diagnosis.
• Hepatobiliary iminodiacetic acid (HIDA) scans demonstrate no filling of the gallbladder in gallbladder volvulus.
• Persisting cholecystitis symptoms in the absence of gallstones can be indicative of gallbladder volvulus.

## 1 Introduction

Gallbladder torsion, alternatively named gallbladder volvulus, is a rare cause of right upper quadrant pain and an important differential to consider in cases of acute surgical abdomen [1]. A recent review suggested that only about 500 cases have been described since the first case was identified in 1898 [2,3]. Gallbladder torsion occurs when the organ twists around its long axis, to the point of occluding vascular flow and bile drainage [4]. Torsion can occur in either clockwise or anticlockwise direction and is considered a complete torsion if the rotation is >180 degrees [4]. Gallbladder torsion can occur at any age but is most commonly seen in 60−80-year olds with a female to male ratio of 3:1 in adults [[5], [6], [7]]. While most cases of torsion are likely idiopathic, some suggested predisposing factors include weight loss, loss of visceral fat, liver atrophy, and certain anatomical variations [4,8,9]. The symptoms of gallbladder volvulus mimic acute cholecystitis and the diagnosis is challenging to make based on pre-operative imaging. However, a delay in diagnosis can result in gangrene of the gallbladder and sepsis. In this article, we report the case of a 77-year-old female with a gallbladder volvulus, who underwent four different imaging modalities that all reported acalculous cholecystitis. She was managed at a tertiary care teaching hospital in Saskatchewan by the acute care surgical team. Her procedure was performed by a fellowship trained general surgeon with over 25 years of operative experience. The work has been reported in line with the SCARE 2020 criteria [10].

## 2 Case

**Fig. 1:**
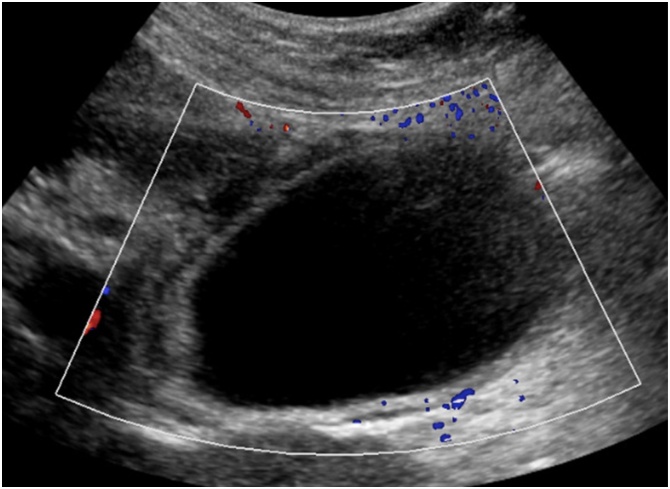
Ultrasound of a gallbladder in a 77-year-old female demonstrating 3 mm of wall thickness, distention, mild pericholecystic fluid, and no cholelithiasis. Intraoperatively, she was discovered to have a gallbladder volvulus.

**Fig. 2:**
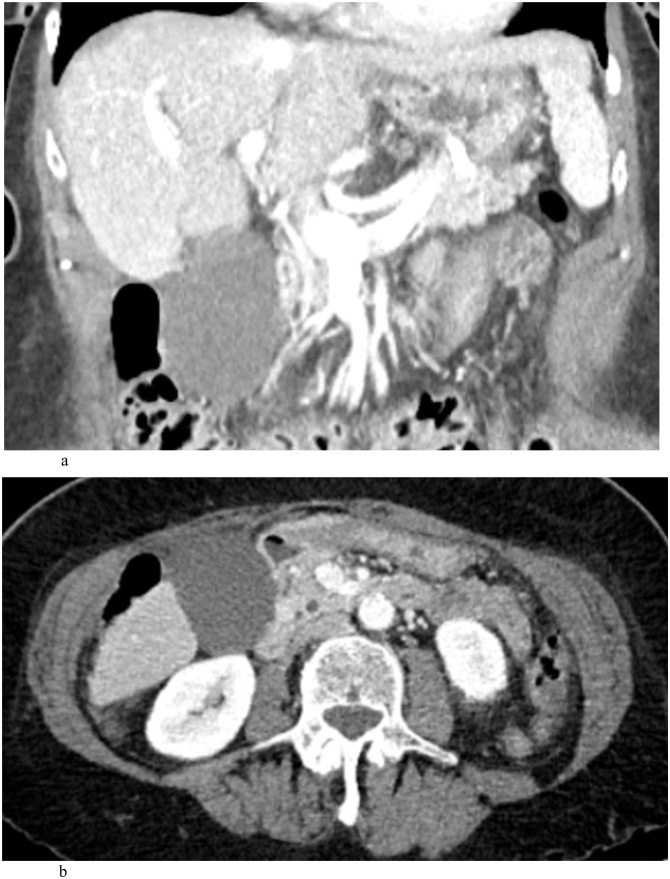
a) Contrast enhanced coronal CT of a gallbladder in a 77-year-old female demonstrating distention and mild pericholecystic fluid, without additional features of cholecystitis. The gallbladder was not enhancing on the scan, and the cystic artery could not be identified. Intraoperatively, she was discovered to have gallbladder volvulus. b) Axial CT of a gallbladder in a 77-year-old female demonstrating distention and mild pericholecystic fluid, without additional features of cholecystitis.

**Fig. 3:**
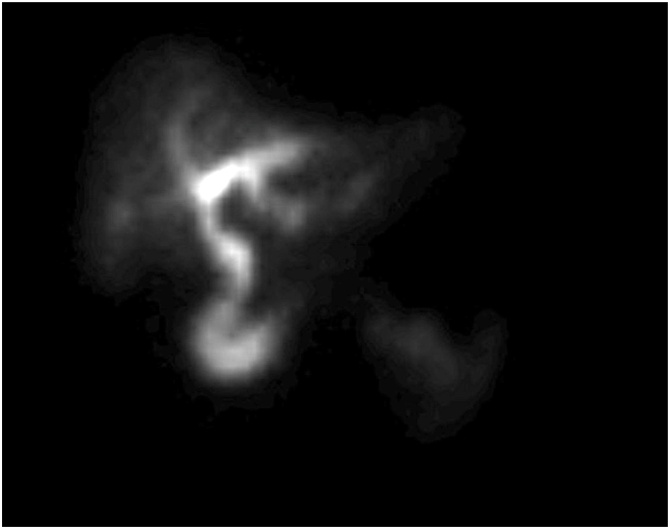
HIDA scan of a 77-year-old female who was discovered to have gallbladder volvulus. There is normal filling of the intrahepatic and extrahepatic bile ducts. The gallbladder and cystic duct do not fill with any contrast.

**Fig. 4:**
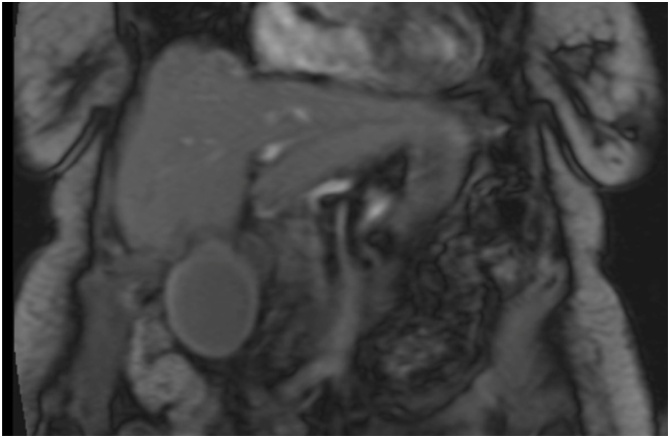
MRCP of a gallbladder showing distention, wall thickness at 3 mm, without gallstones. The extra- and intrahepatic bile ducts are within normal limits.

A 77-year-old woman presented with a 1-day history of constant, sharp, right upper quadrant and epigastric abdominal pain, associated with multiple episodes of non-bloody emesis. She did not identify any triggers for the pain, and specifically stated that the pain is not worse post-prandially. She endorsed a history of recurrent episodes of similar pain over the past several months that were 10–15 min s in duration and always self-resolving. Her past medical, surgical, and family history were non-contributory. She was on no home medications. Laboratory findings were initially unremarkable, but upon re-draw the next day, white blood cell count and C-reactive protein (CRP) were mildly elevated. An ultrasound of the abdomen showed a distended gallbladder with mild pericholecystic fluid, a positive sonographic Murphy’s sign, but no cholelithiasis or extrahepatic bile duct dilatation (Fig. 1). Given these findings, a diagnosis of cholecystitis was questioned, and a lack of cholelithiasis prompted a CT scan of the abdomen and pelvis for further confirmation. The CT also showed a distended gallbladder with mild pericholecystic fluid without additional findings of cholelithiasis, malignancy, or other biliary pathology (Fig. 2). The gallbladder was not enhancing in the CT scan, and the cystic artery was not identified either (Fig. 2a.). At this point, the patient was admitted under the general surgery service with a presumed diagnosis of acalculous cholecystitis. However, she had no other medical comorbidities that are usually found in patients with acalculous cholecystitis. Due to diagnostic uncertainty, a hepatobiliary iminodiacetic acid (HIDA) scan was obtained that showed normal hepatic uptake and excretion, normal excretion in bile ducts, but an ejection fraction of the gallbladder could not be assessed because of incomplete filling. In fact, there was no contrast uptake in the gallbladder, suggesting a complete occlusion of the cystic duct from an unknown cause (Fig. 3). Following the HIDA, an MRCP was obtained to rule out other potential causes of gallbladder inflammation. Similar to the US and CT, the MRCP demonstrated gallbladder distension, gallbladder wall thickening at 3 mm, mild pericholecystic fluid, without cholelithiasis (Fig. 4). Imaging findings in all studies were interpreted as acalculous cholecystitis. The patient was initially treated with 5 days of intravenous ceftriaxone and metronidazole. Despite adherence to the antibiotic regimen there was no improvement in symptoms. Eventually, due to the lack of resolution with antibiotics, a decision was made to proceed with cholecystectomy. A percutaneous decompression tube was not pursued because the patient was otherwise healthy and a good operative candidate.

**Fig. 5:**
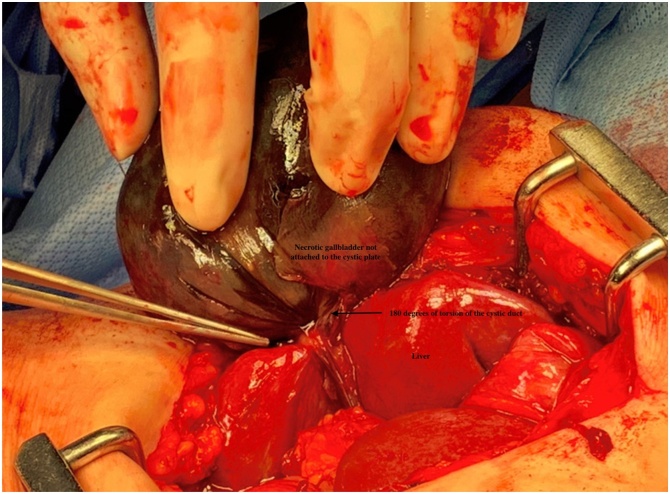
Intraoperative photograph of a gallbladder volvulus in a 77-year-old female patient, demonstrating 180 degrees of torsion of the cystic duct with gangrenous changes. The necrotic gallbladder, liver, and the twisted cystic artery have been labeled.

The procedure was initiated laparoscopically, but there were dense adhesions in the right upper quadrant not allowing for safe visualization of the gallbladder, which resulted in conversion to an open cholecystectomy. Intraoperatively, the gallbladder was found to be 180 degrees torted in an anticlockwise direction. The torsion caused a complete occlusion of the cystic duct and cystic artery resulting in a gangrenous and necrotic gallbladder (Fig. 5). In retrospect, the occlusion of the cystic duct on HIDA was probably the result of a complete torsion of the cystic duct. The gangrenous gallbladder was then removed in a retrograde fashion. The final pathology reported gangrenous cholecystitis with hemorrhage and necrosis at the cystic duct margin. The patient had complete resolution of pain and nausea on post-operative day 1 and was appropriately discharged by post-operative day 2. At her 6 month virtual follow-up, she was content with the overall outcome and was completely symptom free.

## 3 Discussion

Gallbladder torsions have been studied and described through case reports. The statistical sensitivity and specificity of clinical, laboratory and imaging findings have not been determined, likely due to paucity of cases [11]. However, based on the previous reports, it appears that the most common symptoms of gallbladder torsion are right upper quadrant pain, with nausea and vomiting [7,[12], [13], [14]]. Laboratory findings are nonspecific and may include elevated white blood cell count and C-reactive protein, with liver enzymes and bilirubin in the normal range [12], [13], [14]]. Imaging techniques used for diagnosing gallbladder volvulus usually include ultrasonography and cross-sectional imaging (CT and MRI), with nuclear medicine and endoscopic procedures used less frequently [4,15]. Although only 10 % of cases of torsion have ever been diagnosed pre-operatively, certain radiological features have been established via case reports. On ultrasonography, a ‘floating gallbladder sign’ or ‘cystic duct knot’ sign should raise suspicion for torsion [4,[11,[15], [16], [17]]. More specifically, if the ultrasound suggests a large anteriorly floating gallbladder without gallstones and a conical appearance of the neck, this can be suggestive of torsion. A v-shaped tapering of the cystic duct or swirling of the cystic vessels can also be seen on ultrasound. Lastly, thumbprinting of the gallbladder can be suggestive of gangrenous changes, which should also prompt urgent laparoscopy to rule out a volvulus. On CT, the ‘whirl sign’ indicates twisting of the cystic vessels. Finally, the HIDA scan may indicate a ‘bull’s eye sign with a fusiform CBD,’ which is another feature of gallbladder volvulus. Although these specific radiological features are established in case reports, the most common imaging findings are gallbladder distension, wall thickening, and pericholecystic inflammatory changes, which are not specific for torsion [4,15]. This is why almost all gallbladder torsions are typically diagnosed intra-operatively [11,13,14].

In this patient, it was unresolving pain on day 5 of intravenous antibiotics that prompted operative management, but if gallbladder torsion was suspected, a cholecystectomy might have been pursued sooner. In retrospect, cystic duct occlusion on HIDA with a lack of gallstones on cross-sectional imaging should have increased suspicion for gallbladder torsion. However, the patient had no specific signs of torsion on imaging, such as whirling, floating, thumbprinting, bull’s eye, or the cystic duct knot sign.

Through this report, we recommend a high index of suspicion for a gallbladder volvulus in elderly female patients admitted with cholecystitis who have no resolution with conservative management, and also have imaging findings of cystic duct occlusion on HIDA without gallstones on MRCP or US.

## 4 Conclusion

The differential diagnoses of acalculous acute cholecystitis should include gallbladder volvulus. In a patient with persistent symptoms of right upper quadrant pain, nausea, and vomiting in the absence of gallstones, gallbladder volvulus must be a strong consideration. The findings of incomplete uptake in the cystic duct on HIDA and MRCP may suggest secondary causes of cystic duct occlusion, such as torsion of the gallbladder, but the diagnosis is essentially a clinical diagnosis.

## Declaration of Competing Interest

The authors report no declarations of interest.

## Funding

The publication of this article was funded by the Department of Surgery, University of Saskatchewan.

## Ethical approval

The study was exempt from the Research Ethics Board (REB) at the University of Saskatchewan as it is a case report, and the study does not influence the care of the patient.

## Consent

Formal consent was obtained from the patient for the publication of this case report and any accompanying images.

## Author contribution

All three authors were part of the circle of care for this patient while in hospital.

Dr. Zarrukh Baig (General Surgery) is the primary author of the article.

Dr. Vladimir Ljubojevic (Radiology) interpreted the imaging findings, formatted the pictures, and helped with the writing of the manuscript. Dr. Francis Christian is the primary investigator, the primary surgeon in the care of this patient, and helped with the study concept.

## Registration of research studies

Not applicable.

## Guarantor

Dr. Francis Christian and Dr. Zarrukh Baig are the guarantors of this publication and accept full responsibility for the work and/or the conduct of this study.

## Provenance and peer review

Not commissioned, externally peer-reviewed.
